# Data for global power demand and solar PV output matching

**DOI:** 10.1016/j.dib.2018.06.054

**Published:** 2018-06-26

**Authors:** Peter D. Lund

**Affiliations:** Aalto University, School of Science, PO Box 15100, FI-00076 AALTO, Espoo, Finland

## Abstract

Increasing use of solar energy necessitates better data sets for analyzing matching of solar photovoltaic output and power demand. Data source information presented in this article is useful to analyze the self-consumption rates of photovoltaic systems on global scale. The data is provided in figure format. The time resolution is basically one hour, but 1-min data is also included. The geographical range of selected sites is lat. 26–60 deg. (Europe, Asia, Latin-America). The power demand data ranges from a single household to national scale. Both measured and simulated data are included. The data sets are linked to a recent article by Lund [1].

**Specifications Table**

**Table:** 

Subject area	Renewable Energy
More specific subject area	Solar Energy
Type of data	Figures
How data was acquired	Simulation, references, internet databases
Data format	Analyzed, raw
Experimental factors	Not applicable
Experimental features	Not applicable
Data source location	Lat. 26–60 deg. (Europe, Asia, Latin-America)
Data accessibility	Data sources provided here
Related research article	Peter D. Lund, Capacity matching of storage to PV in a global frame with different loads profiles, Journal of Energy Storage 18 (2018) 218–228.

**Value of the data**

• The data is helpful for performance simulations of photovoltaic (PV) systems
• The data is applicable for a range of different PV systems
• The data can be used for planning more effective PV systems

## 1 Data

The dataset of this article provides information on the solar photovoltaic (PV) system output and power demand for selected sites and load types over an entire year used in [1]. The geographical range is lat. 26–60 deg. (Europe, Asia, Latin-America).

Fig. 1, Fig. 2, Fig. 3, Fig. 4, Fig. 5, Fig. 6, Fig. 7, Fig. 8, Fig. 9 show the PV output data. Fig. 10, Fig. 11, Fig. 12, Fig. 13, Fig. 14, Fig. 15, Fig. 16, Fig. 17, Fig. 18, Fig. 19, Fig. 20, Fig. 21, Fig. 22, Fig. 23, Fig. 24, Fig. 25, Fig. 26, Fig. 27, Fig. 28, Fig. 29, Fig. 30, Fig. 31, Fig. 32, Fig. 33, Fig. 34 show the PV output data for Helsinki (Finland) during 1973–1997. Fig. 35, Fig. 36, Fig. 37, Fig. 38, Fig. 39, Fig. 40, Fig. 41, Fig. 42, Fig. 43, Fig. 44, Fig. 45 show the power demand data sets.

**Fig. 1:**
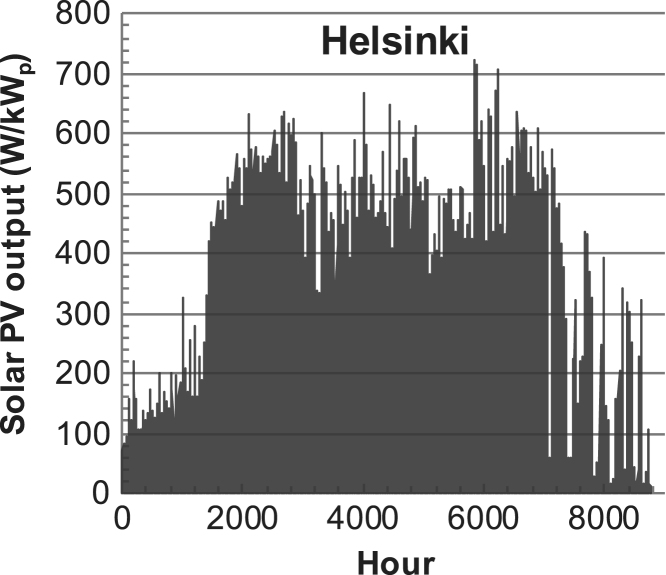
Hourly PV output for Helsinki (Finland) over a year with 1-hour resolution for a 30° tilted surface orientated to the south. The values are scaled to 1 kW_p_ of PV.

**Fig. 2:**
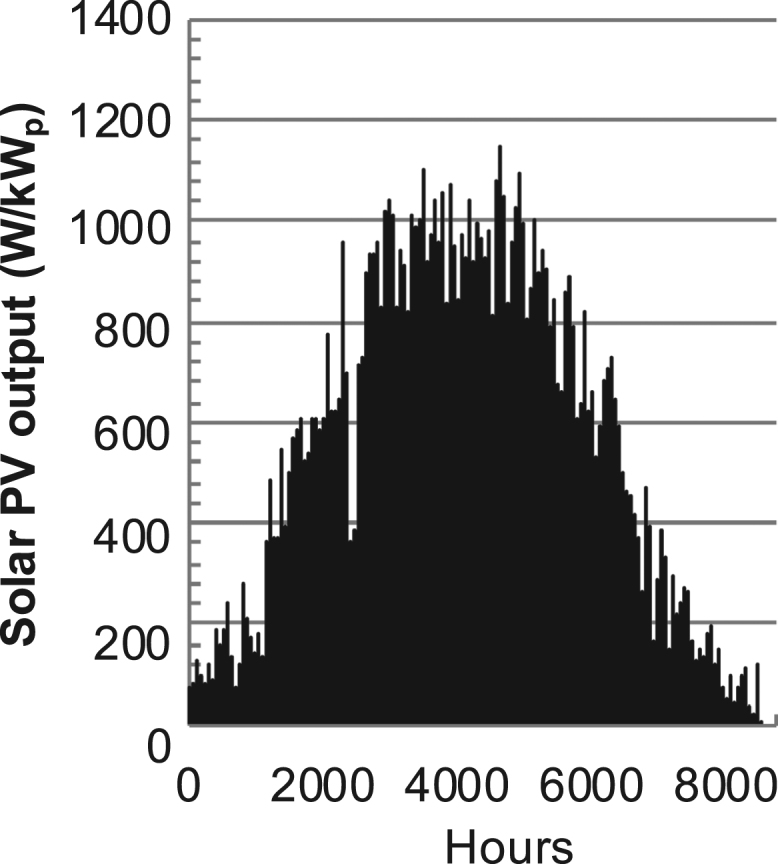
PV output for Helsinki (Finland) over a year with 1-min resolution for a horizontal surface. The values are scaled to 1 kW_p_ of PV.

**Fig. 3:**
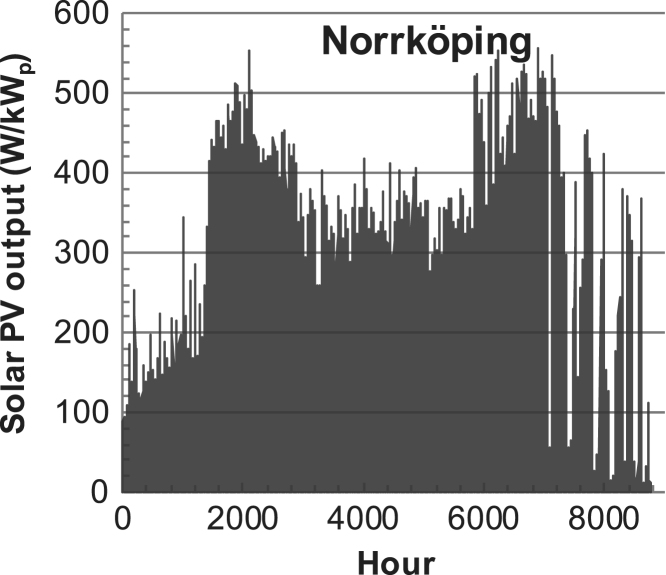
PV output for Norrköping (Sweden) over a year with 10-min resolution for a 30° tilted surface orientated to the south. The values are scaled to 1 kW_p_ of PV.

**Fig. 4:**
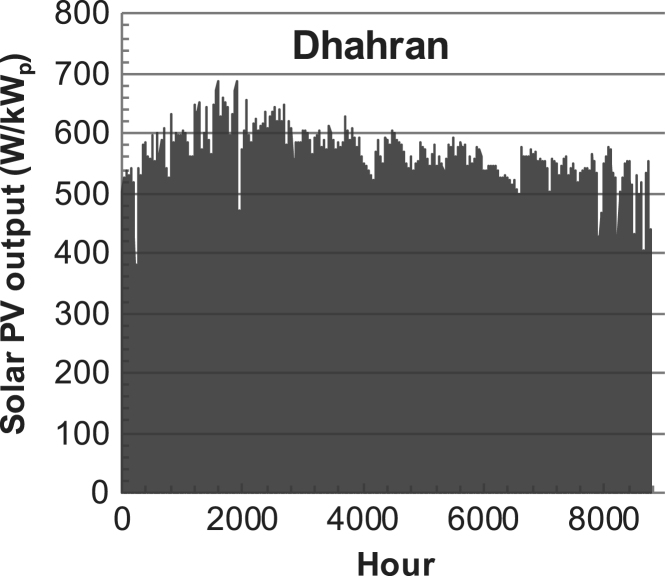
Hourly PV output for Dhahran (Saudi-Arabia) over a year with 1-h resolution for a 30° tilted surface orientated to the south. The values are scaled to 1 kW_p_ of PV.

**Fig. 5:**
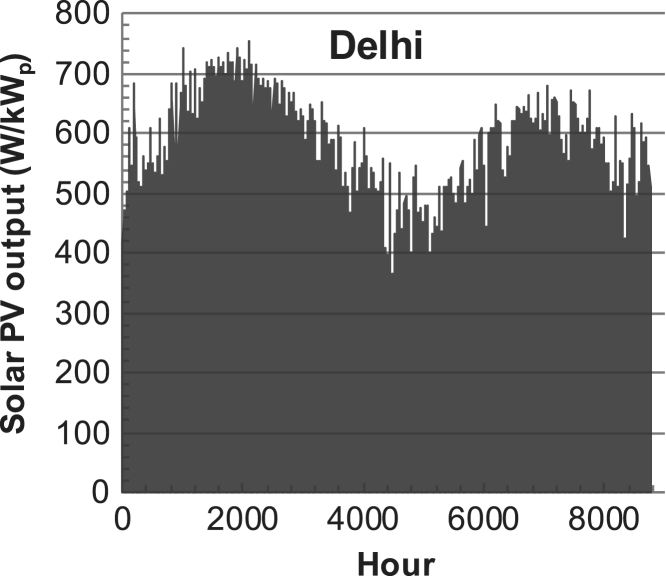
Hourly PV output for Delhi (India) over a year with 1-h resolution for a 30° tilted surface orientated to the south. The values are scaled to 1 kW_p_ of PV.

**Fig. 6:**
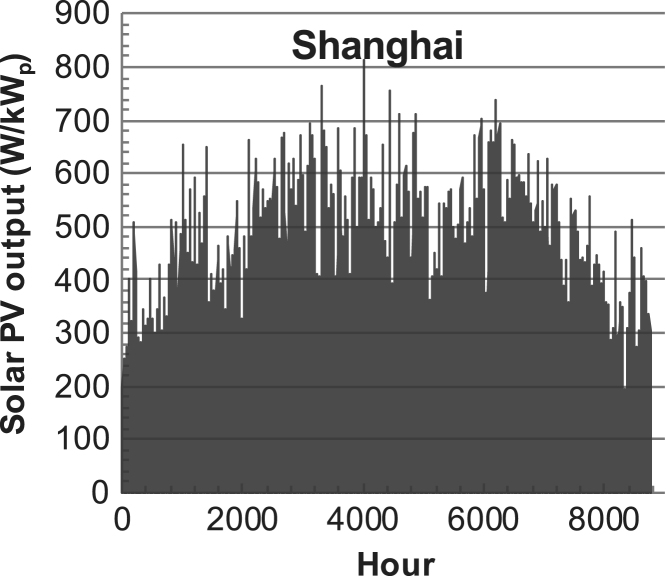
Hourly PV output for Shanghai (China) over a year with 1-h resolution for a 30° tilted surface orientated to the south. The values are scaled to 1 kW_p_ of PV.

**Fig. 7:**
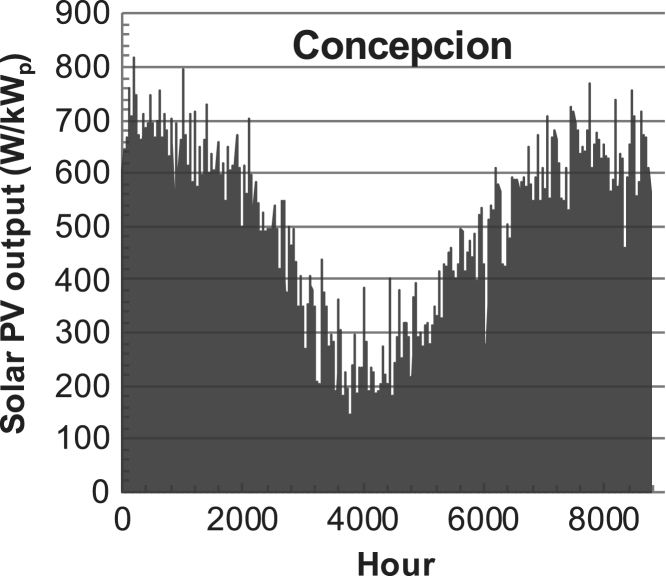
Hourly PV output for Concepcion (Chile) over a year with 1-h resolution for a 30° tilted surface orientated to the south. The values are scaled to 1 kW_p_ of PV.

**Fig. 8:**
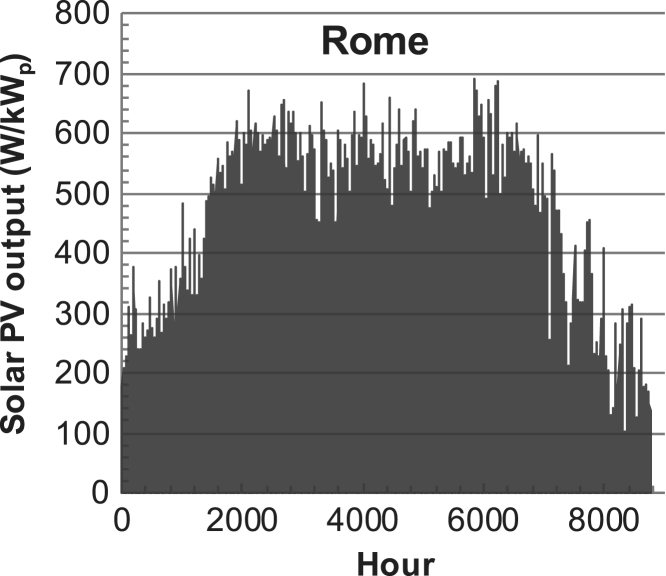
Hourly PV output for Rome (Italy) over a year with 1-h resolution, for a 30° tilted surface orientated to the south. The values are scaled to 1 kW_p_ of PV.

**Fig. 9:**
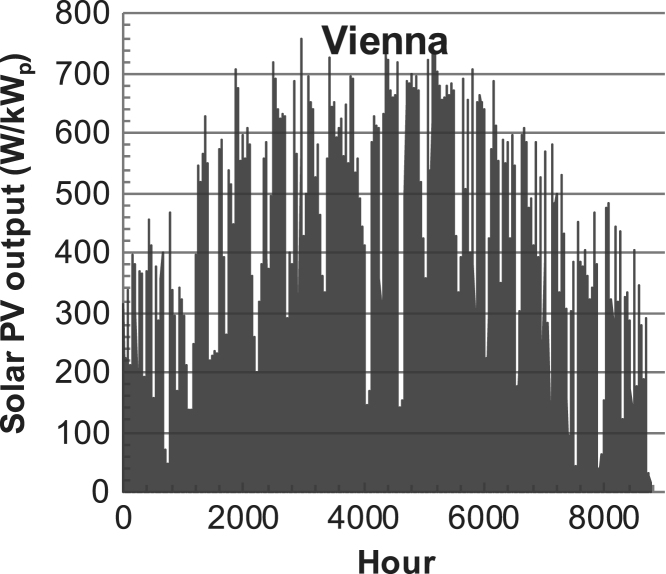
Hourly PV output for Vienna (Austria) over a year with 1-h resolution, for a 30° tilted surface orientated to the south. The values are scaled to 1 kW_p_ of PV.

**Fig. 10:**
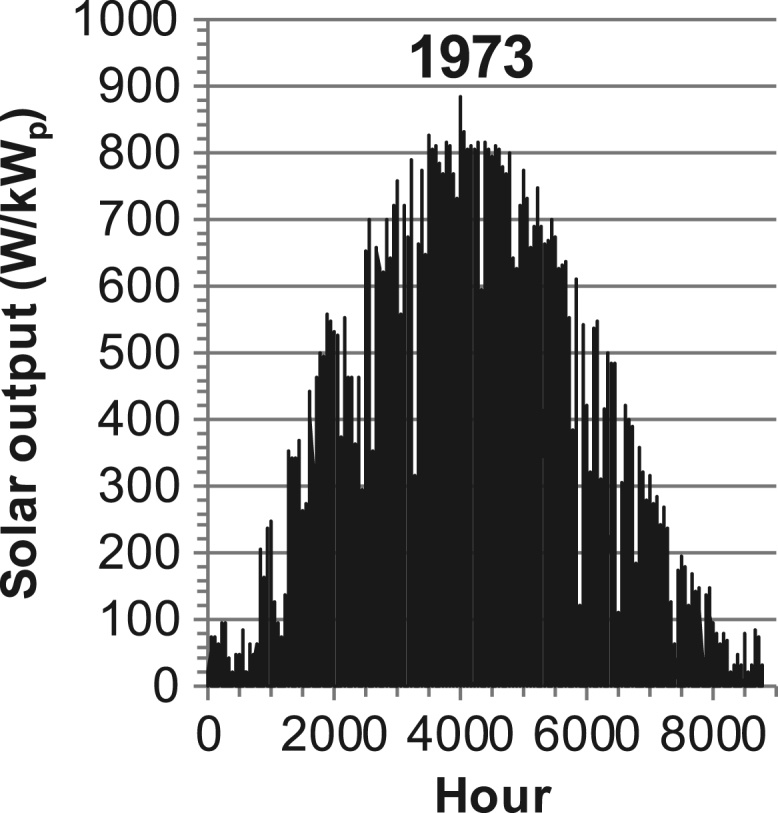
Hourly PV output for Helsinki (Finland) for year 1973 with 1-h resolution, horizontal surface. The values are scaled to 1 kW_p_ of PV.

**Fig. 11:**
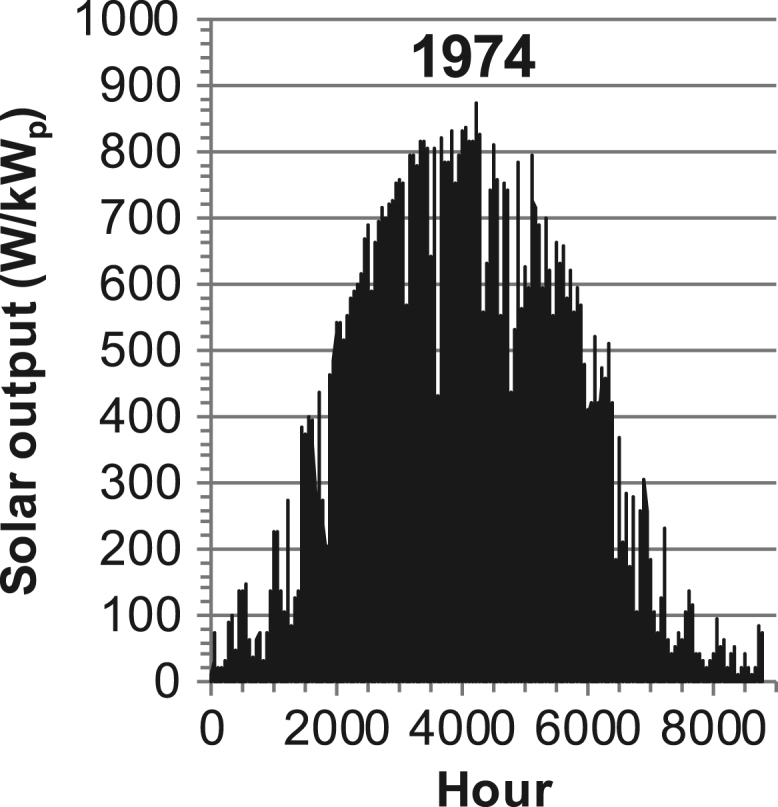
Hourly PV output for Helsinki (Finland) for year 1974 with 1-h resolution, horizontal surface. The values are scaled to 1 kW_p_ of PV.

**Fig. 12:**
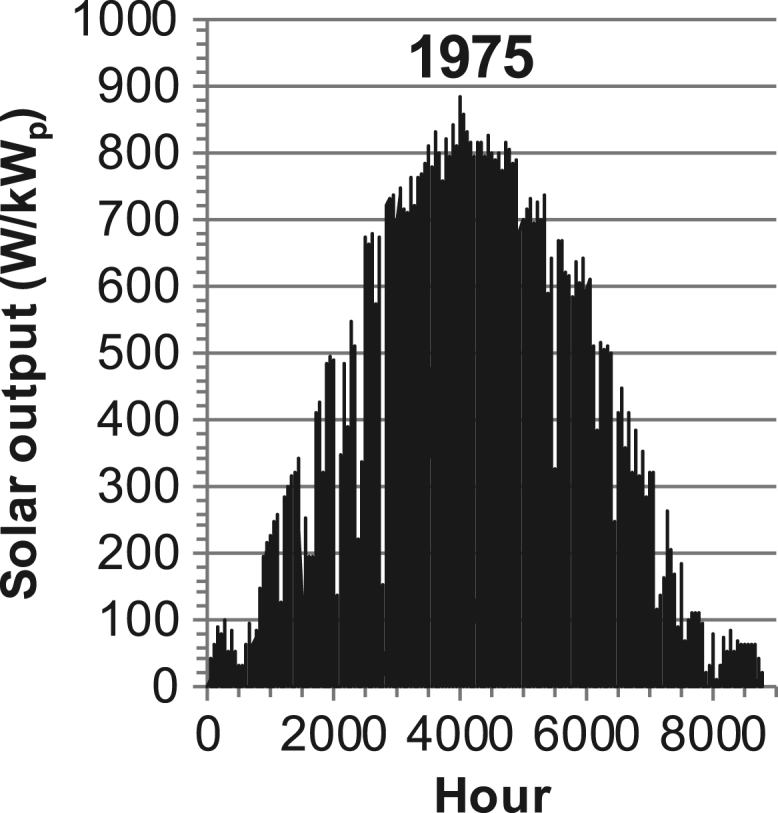
Hourly PV output for Helsinki (Finland) for year 1975 with 1-h resolution, horizontal surface. The values are scaled to 1 kW_p_ of PV.

**Fig. 13:**
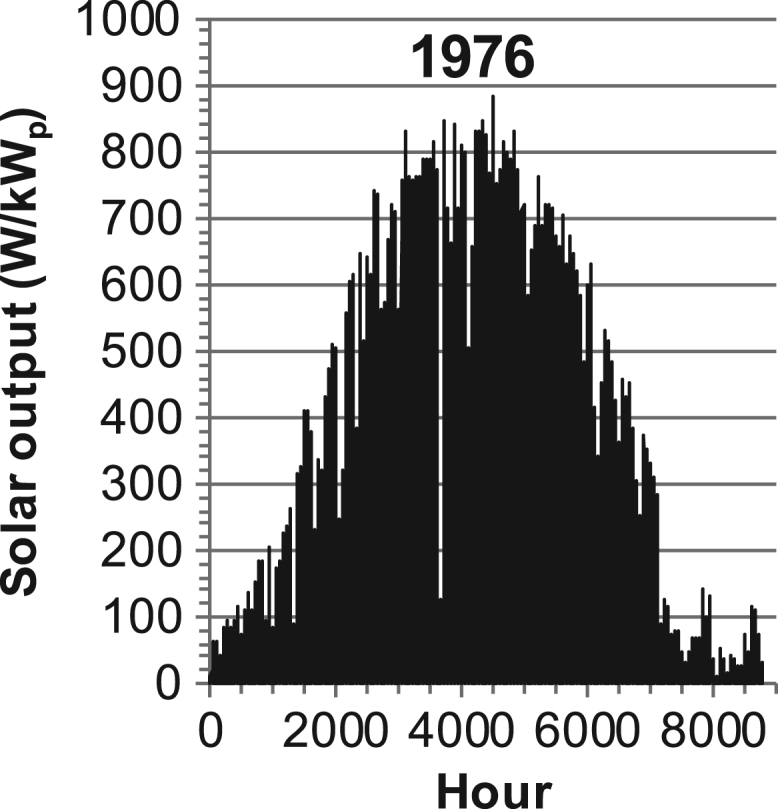
Hourly PV output for Helsinki (Finland) for year 1976 with 1-h resolution, horizontal surface. The values are scaled to 1 kW_p_ of PV.

**Fig. 14:**
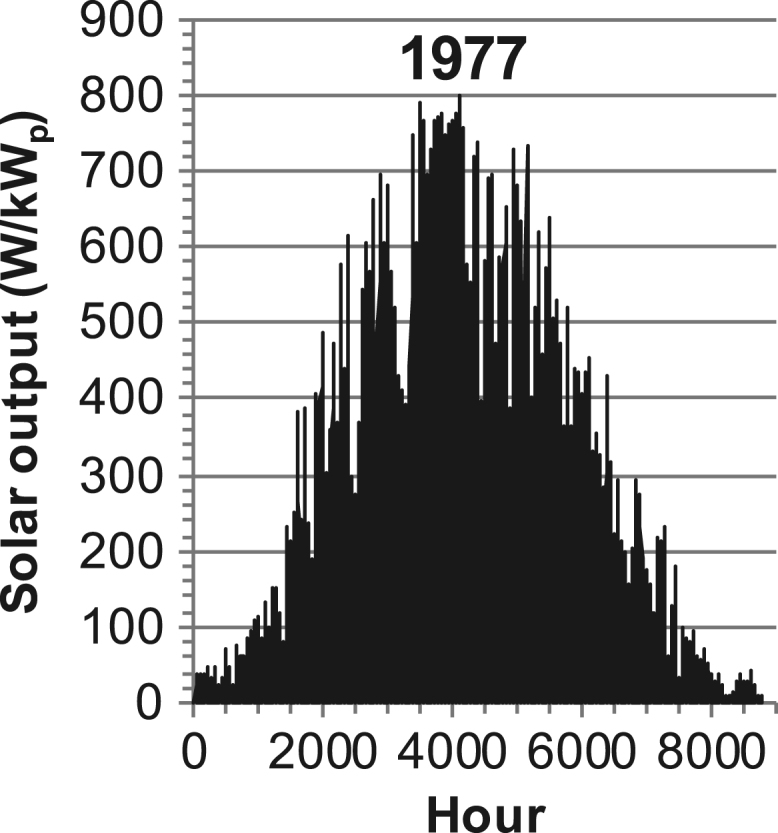
Hourly PV output for Helsinki (Finland) for year 1977 with 1-h resolution, horizontal surface. The values are scaled to 1 kW_p_ of PV.

**Fig. 15:**
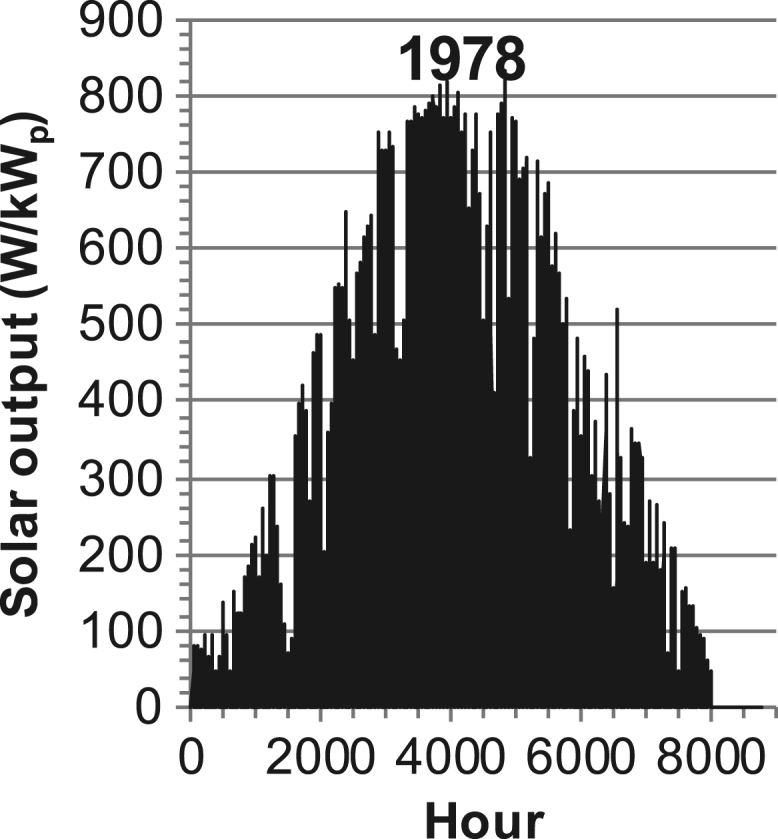
Hourly PV output for Helsinki (Finland) for year 1978 with 1-h resolution, horizontal surface. The values are scaled to 1 kW_p_ of PV.

**Fig. 16:**
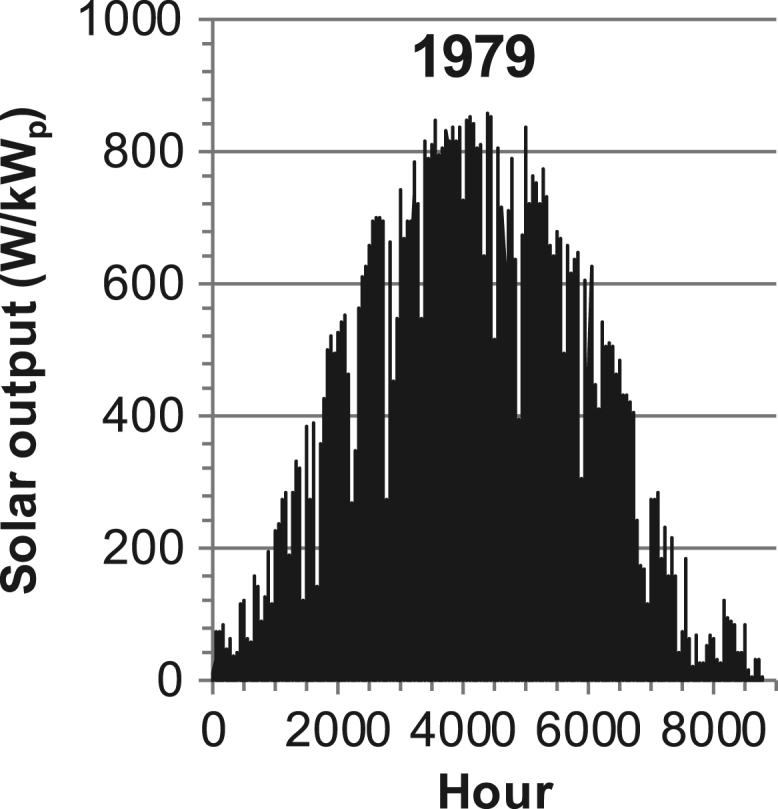
Hourly PV output for Helsinki (Finland) for year 1979 with 1-h resolution, horizontal surface. The values are scaled to 1 kW_p_ of PV.

**Fig. 17:**
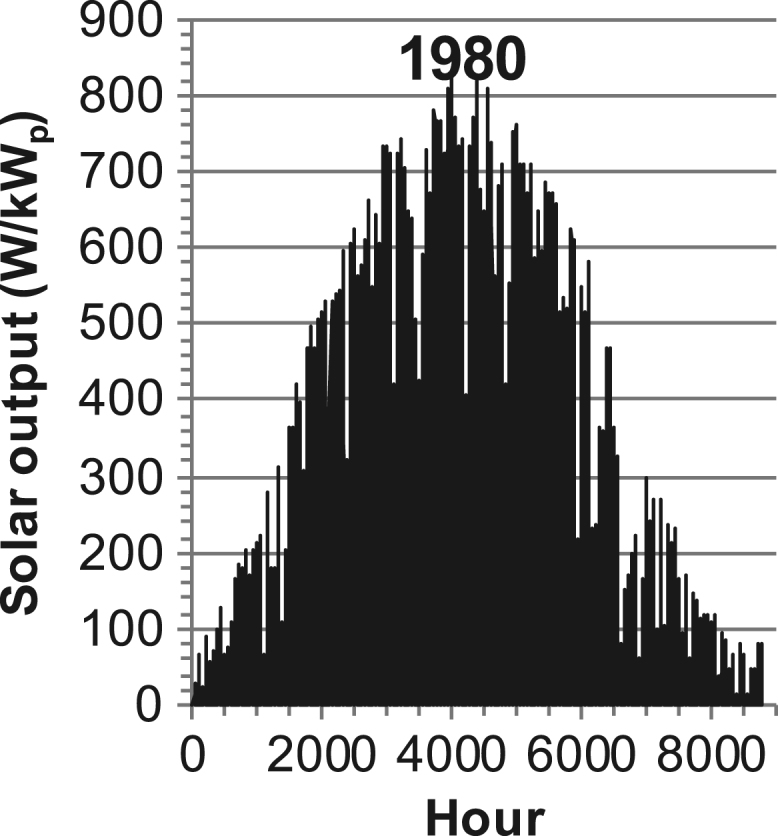
Hourly PV output for Helsinki (Finland) for year 1980 with 1-hour resolution, horizontal surface. The values are scaled to 1 kW_p_ of PV.

**Fig. 18:**
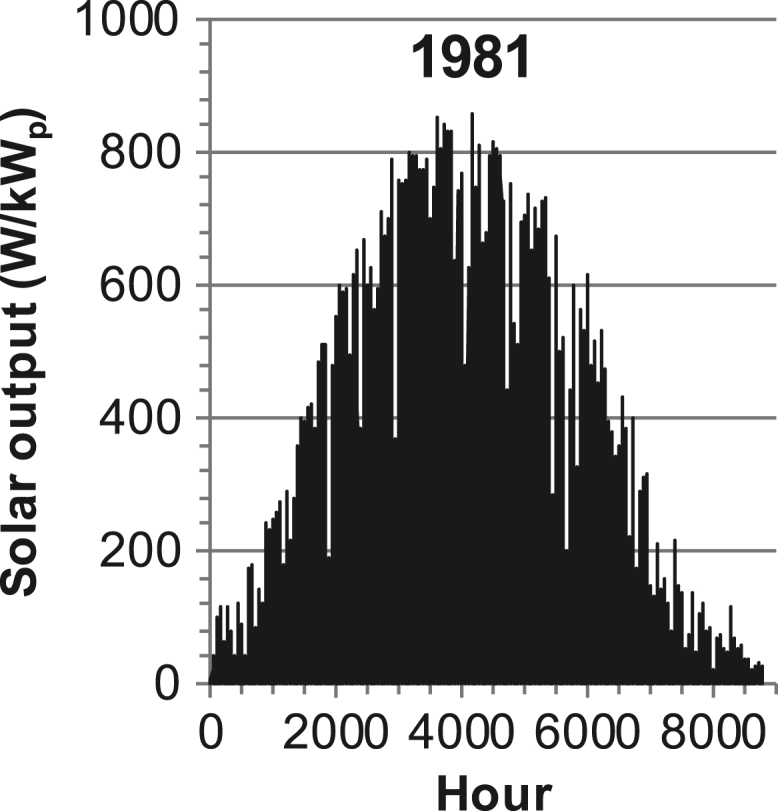
Hourly PV output for Helsinki (Finland) for year 1981 with 1-h resolution, horizontal surface. The values are scaled to 1 kW_p_ of PV.

**Fig. 19:**
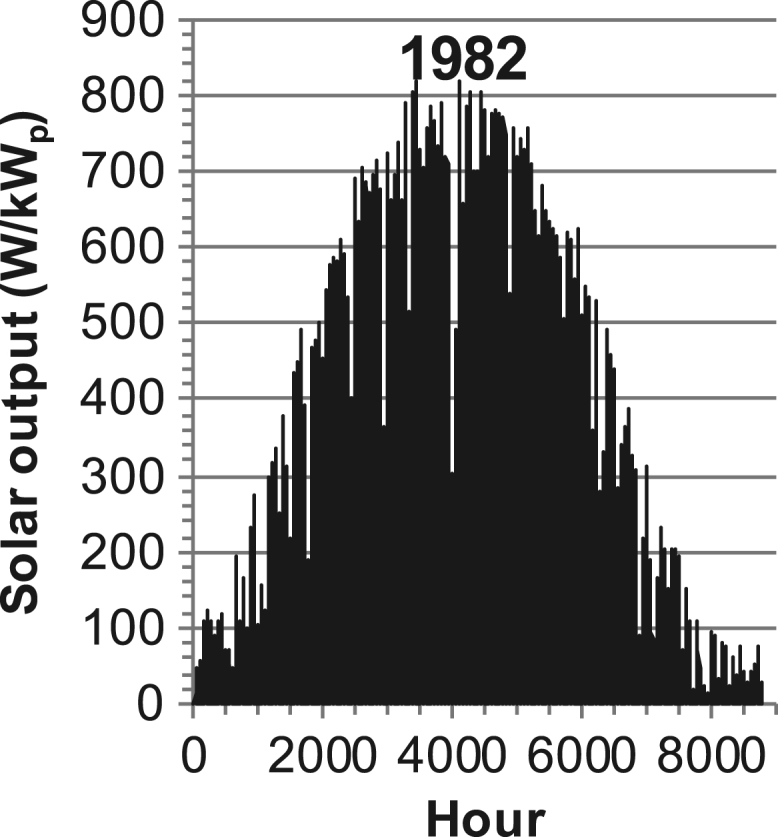
Hourly PV output for Helsinki (Finland) for year 1982 with 1-hour resolution, horizontal surface. The values are scaled to 1 kW_p_ of PV.

**Fig. 20:**
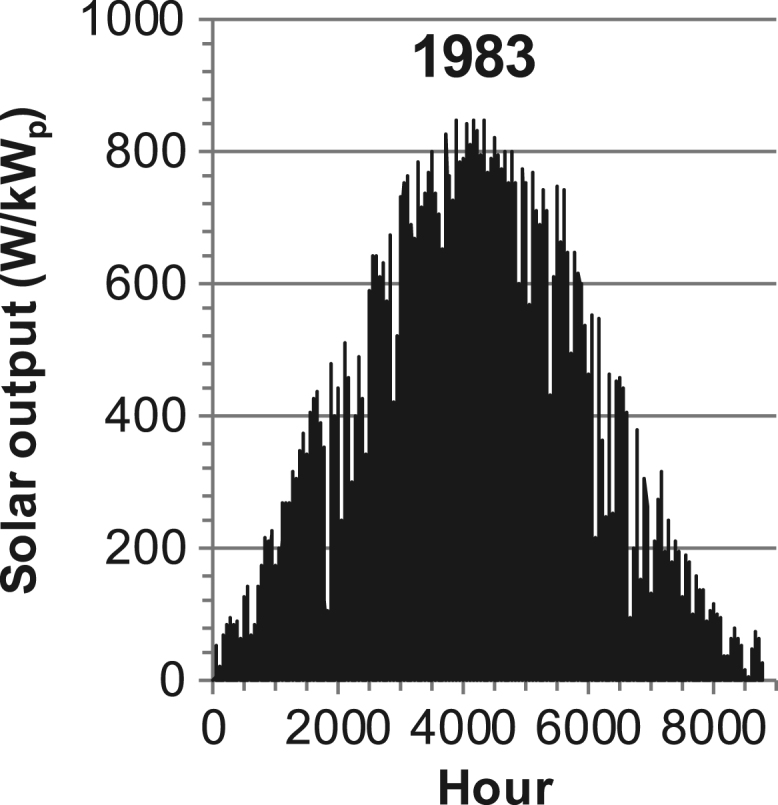
Hourly PV output for Helsinki (Finland) for year 1983 with 1-hour resolution, horizontal surface. The values are scaled to 1 kW_p_ of PV.

**Fig. 21:**
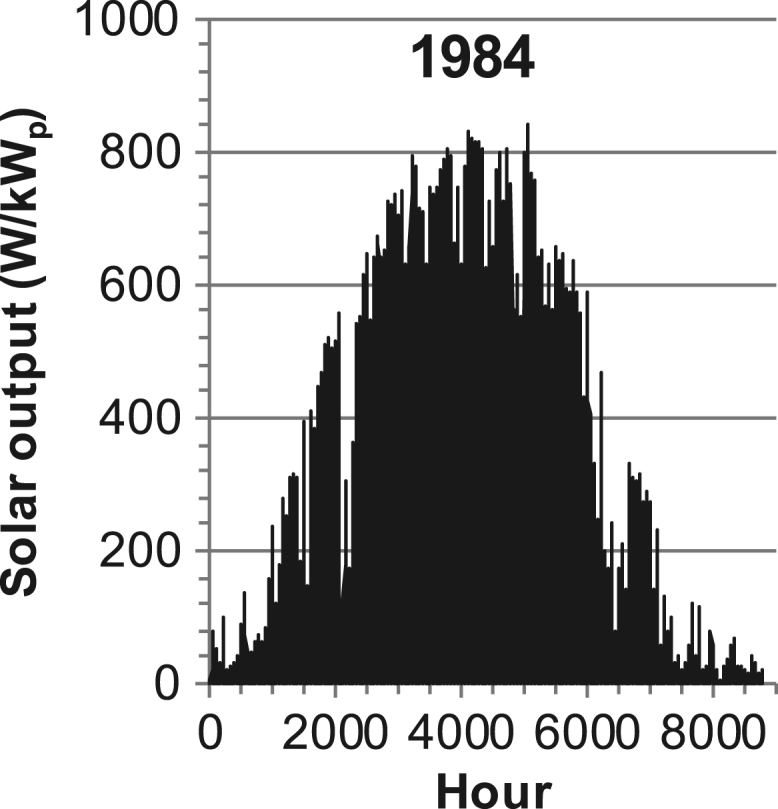
Hourly PV output for Helsinki (Finland) for year 1984 with 1-h resolution, horizontal surface. The values are scaled to 1 kW_p_ of PV.

**Fig. 22:**
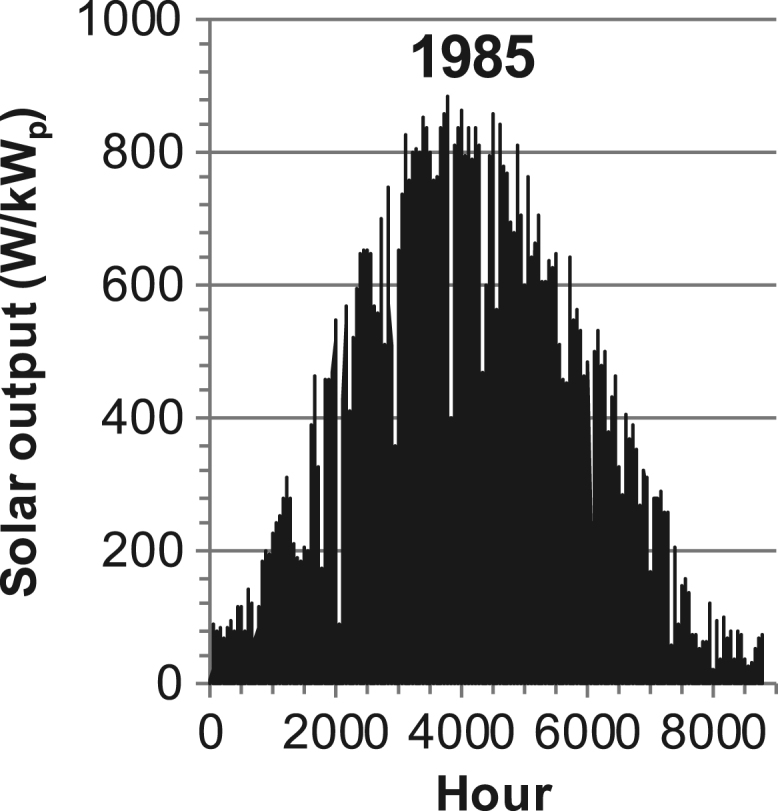
Hourly PV output for Helsinki (Finland) for year 1985 with 1-h resolution, horizontal surface. The values are scaled to 1 kW_p_ of PV.

**Fig. 23:**
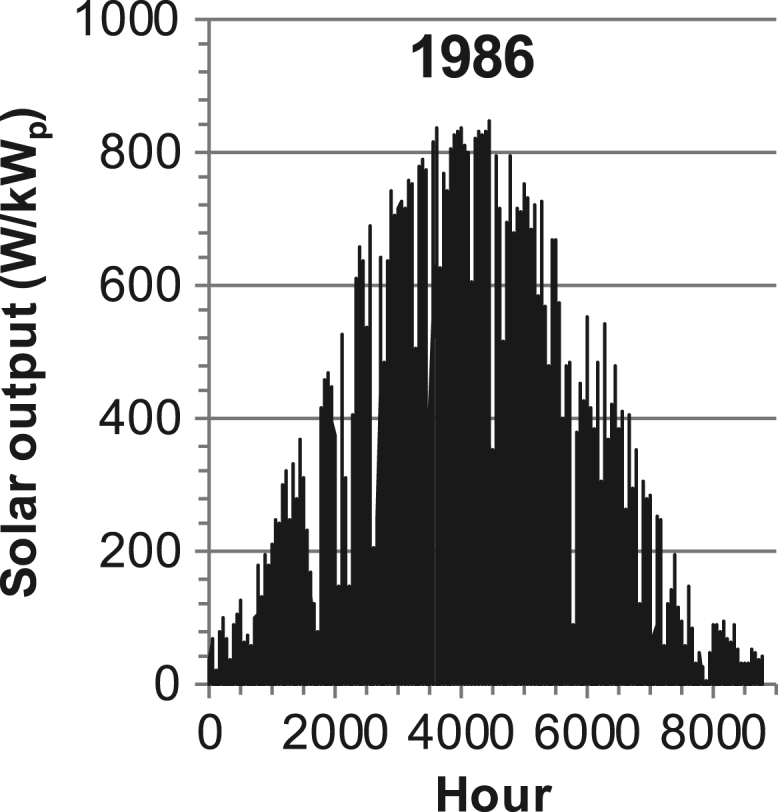
Hourly PV output for Helsinki (Finland) for year 1986 with 1-h resolution, horizontal surface. The values are scaled to 1 kW_p_ of PV.

**Fig. 24:**
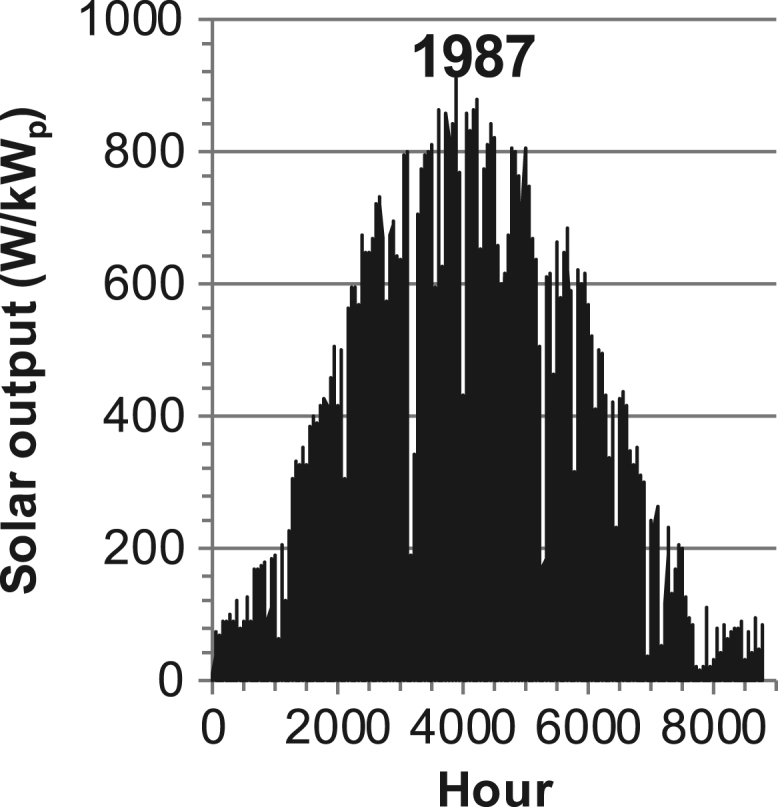
Hourly PV output for Helsinki (Finland) for year 1987 with 1-h resolution, horizontal surface. The values are scaled to 1 kW_p_ of PV.

**Fig. 25:**
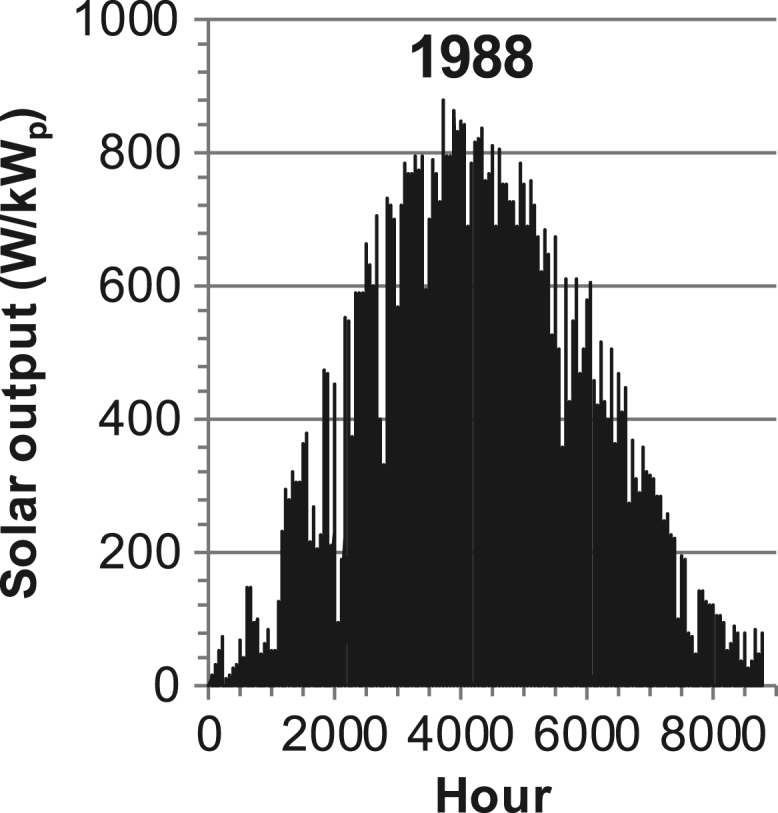
Hourly PV output for Helsinki (Finland) for year 1988 with 1-h resolution, horizontal surface. The values are scaled to 1 kW_p_ of PV.

**Fig. 26:**
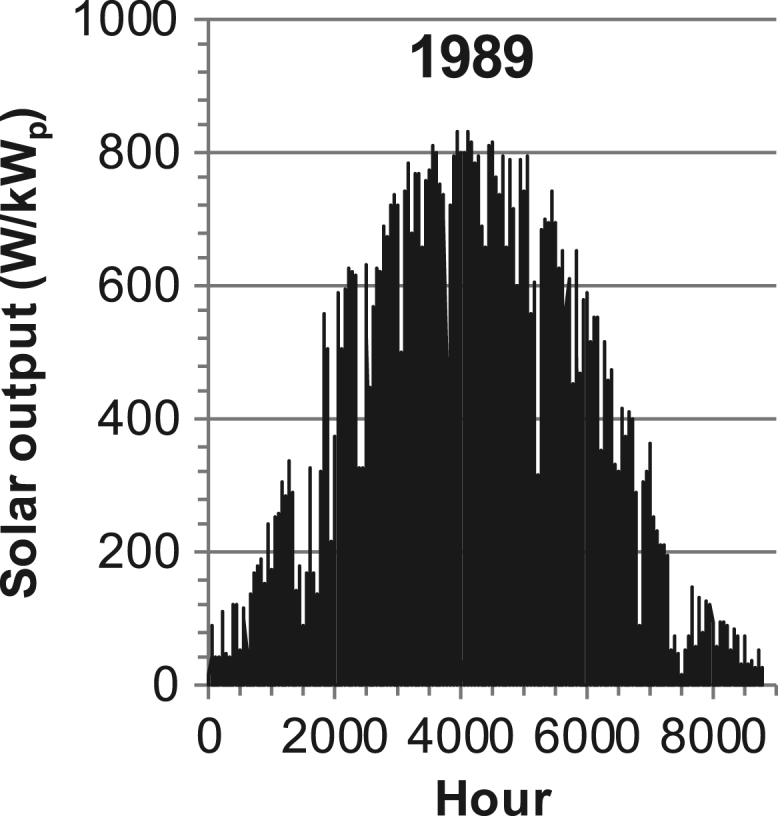
Hourly PV output for Helsinki (Finland) for year 1989 with 1-h resolution, horizontal surface. The values are scaled to 1 kW_p_ of PV.

**Fig. 27:**
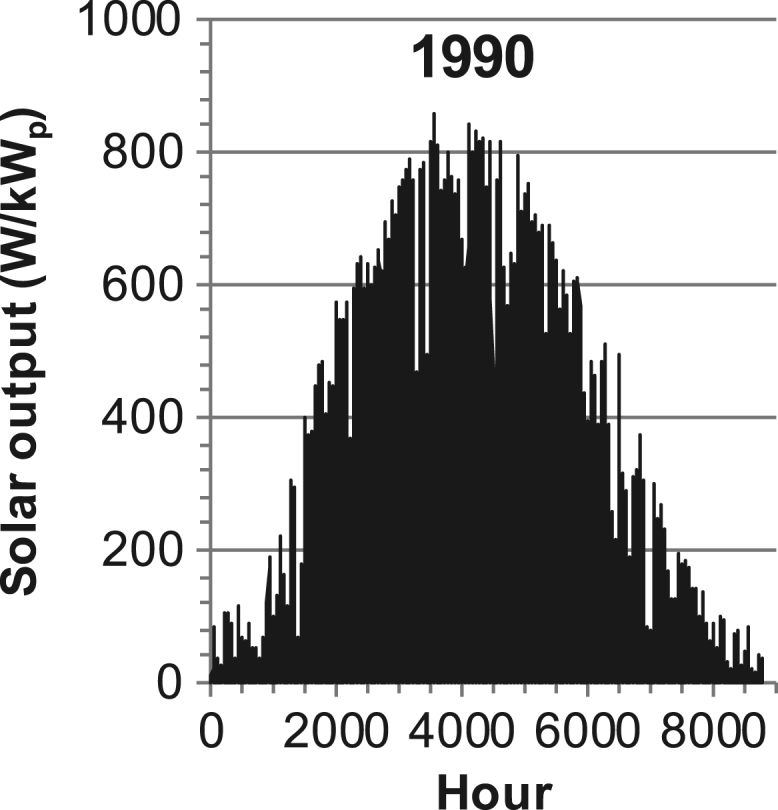
Hourly PV output for Helsinki (Finland) for year 1990 with 1-h resolution, horizontal surface. The values are scaled to 1 kW_p_ of PV.

**Fig. 28:**
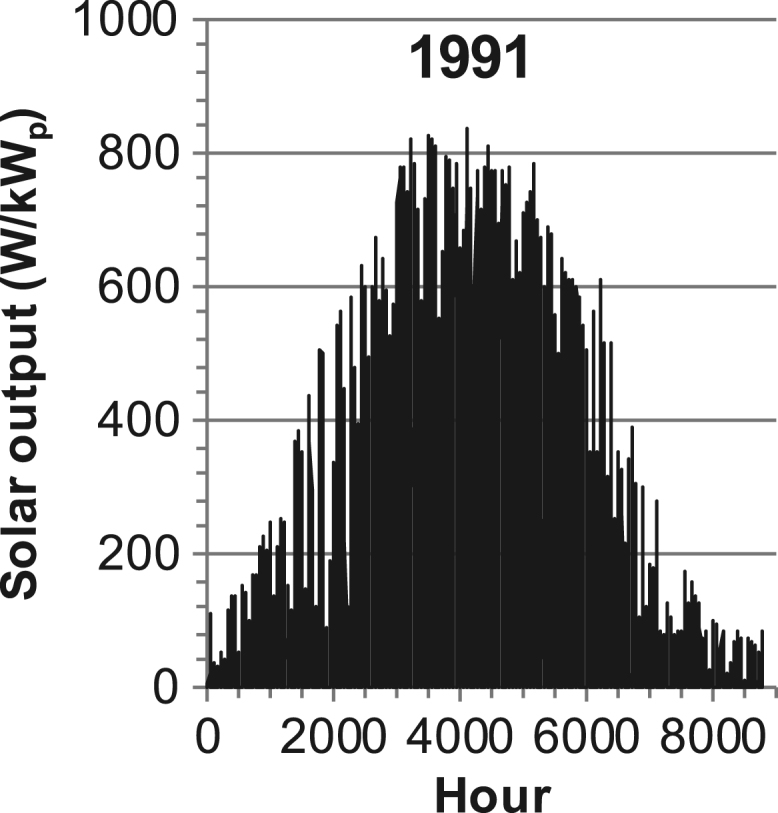
Hourly PV output for Helsinki (Finland) for year 1991 with 1-h resolution, horizontal surface. The values are scaled to 1 kW_p_ of PV.

**Fig. 29:**
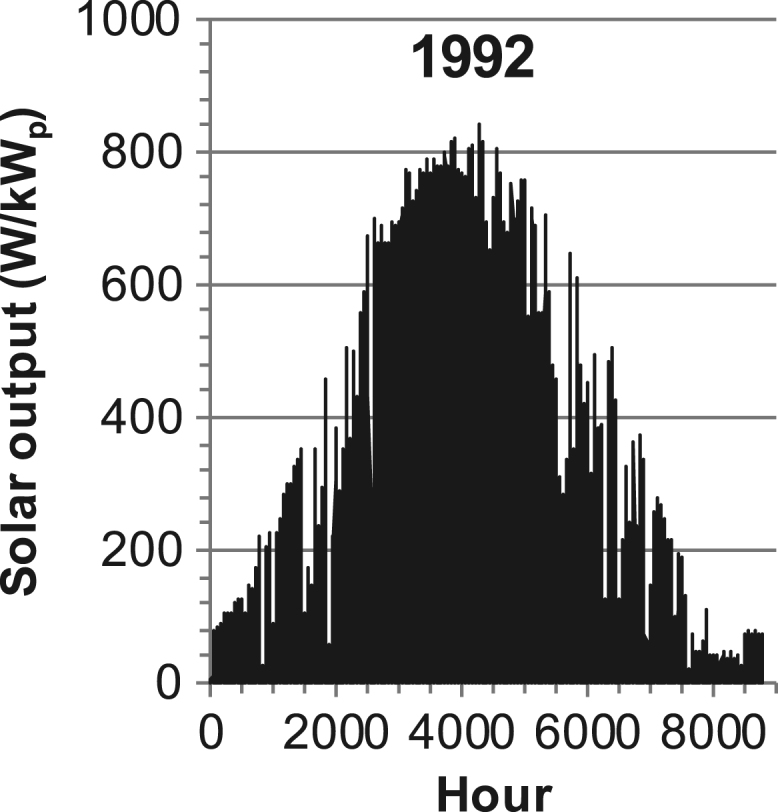
Hourly PV output for Helsinki (Finland) for year 1992 with 1-hour resolution, horizontal surface. The values are scaled to 1 kW_p_ of PV.

**Fig. 30:**
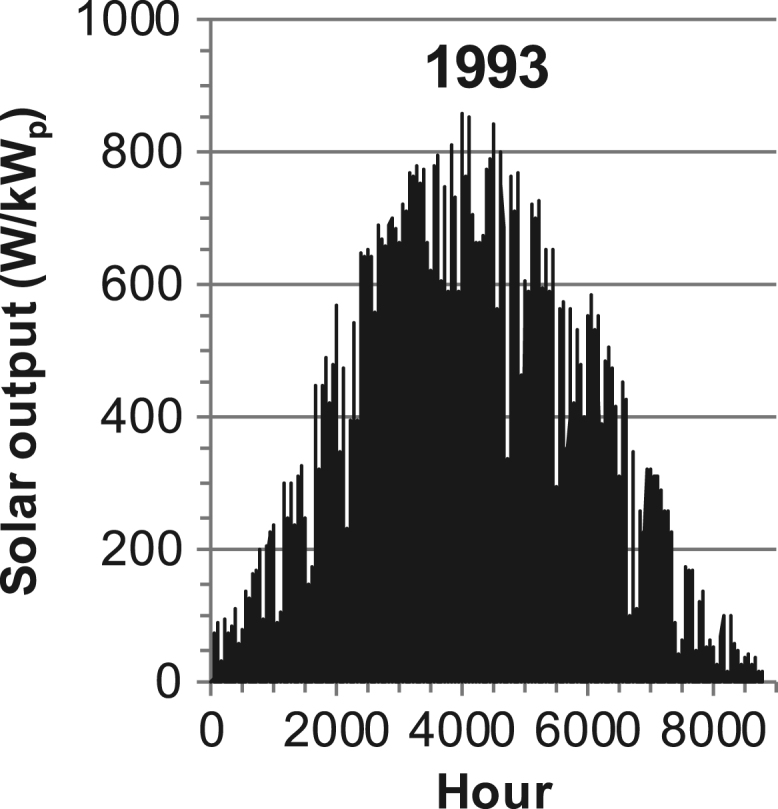
Hourly PV output for Helsinki (Finland) for year 1993 with 1-h resolution, horizontal surface. The values are scaled to 1 kW_p_ of PV.

**Fig. 31:**
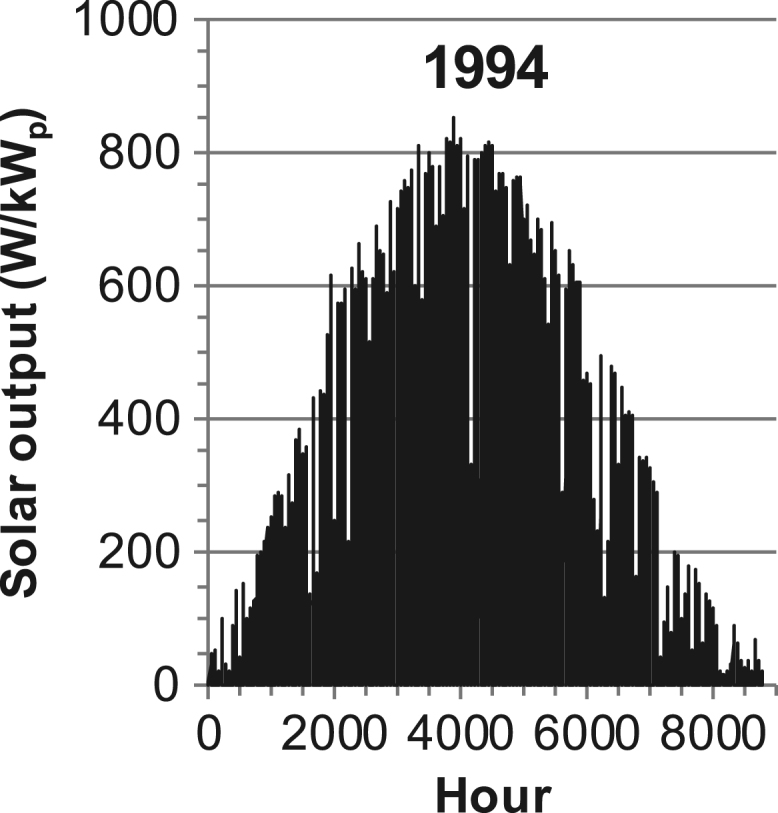
Hourly PV output for Helsinki (Finland) for year 1994 with 1-h resolution, horizontal surface. The values are scaled to 1 kW_p_ of PV.

**Fig. 32:**
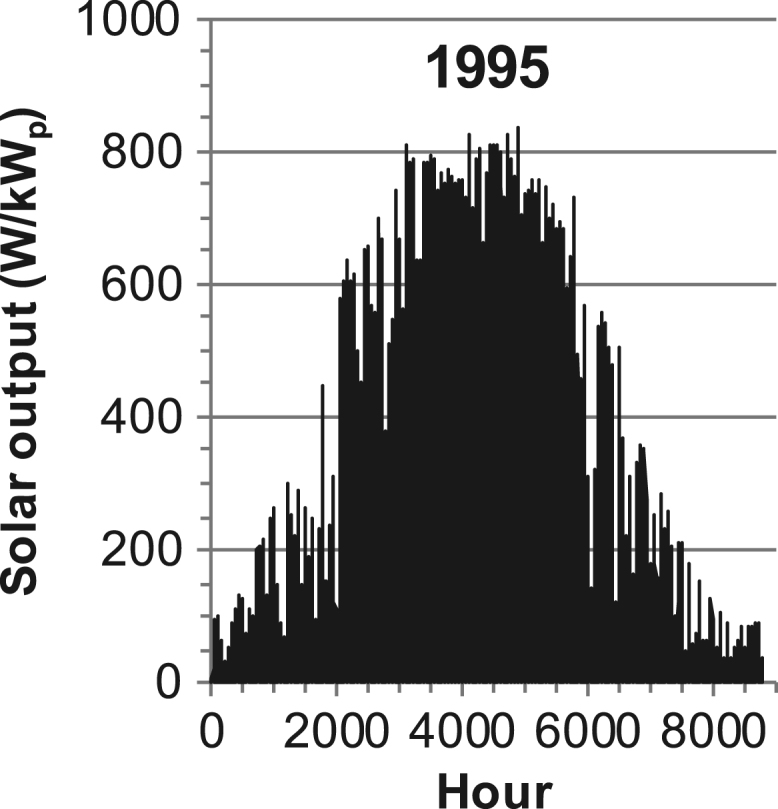
Hourly PV output for Helsinki (Finland) for year 1995 with 1-h resolution, horizontal surface. The values are scaled to 1 kW_p_ of PV.

**Fig. 33:**
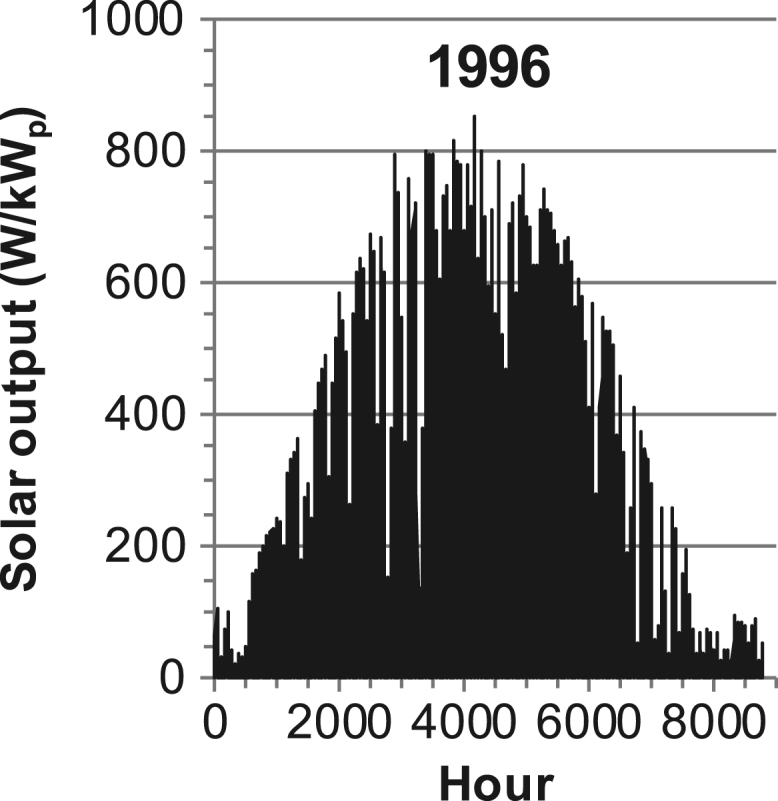
Hourly PV output for Helsinki (Finland) for year 1996 with 1-h resolution, horizontal surface. The values are scaled to 1 kW_p_ of PV.

**Fig. 34:**
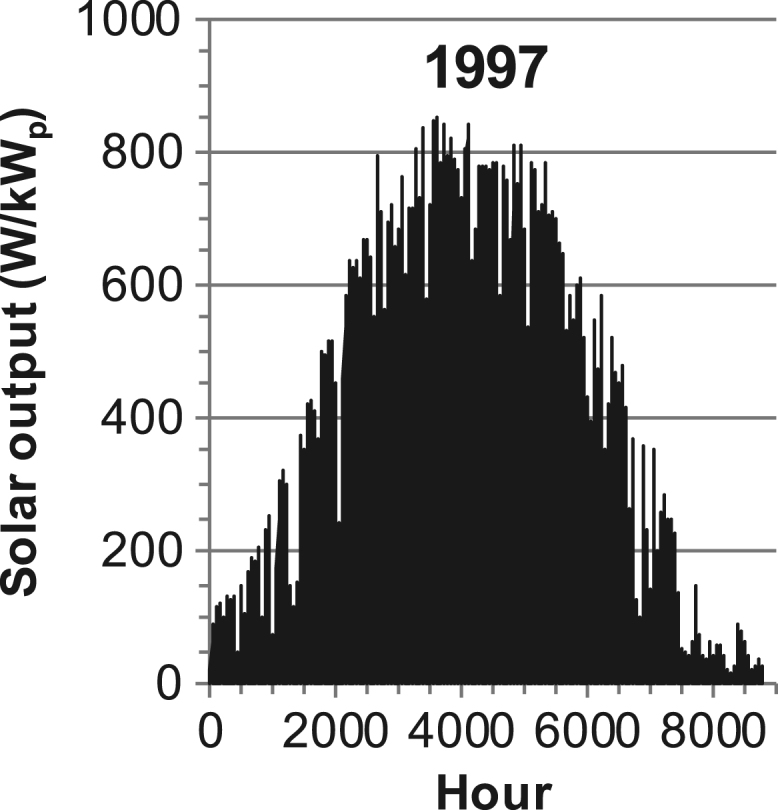
Hourly PV output for Helsinki (Finland) for year 1997 with 1-hour resolution, horizontal surface. The values are scaled to 1 kW_p_ of PV.

**Fig. 35:**
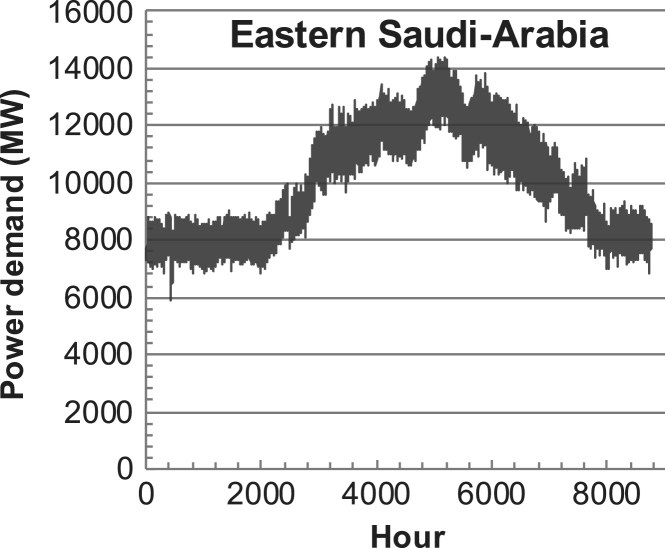
Hourly power demand for Eastern Saudi-Arabia over a year (data with 1-h resolution).

**Fig. 36:**
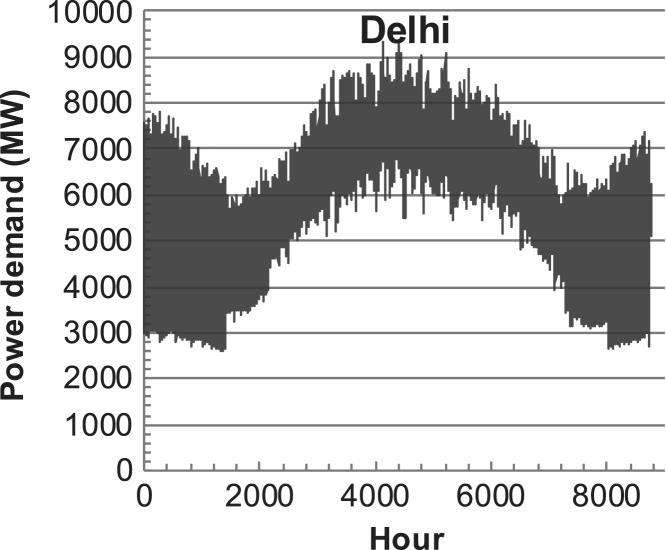
Hourly power demand for Delhi (India) over a year (data with 1-h resolution).

**Fig. 37:**
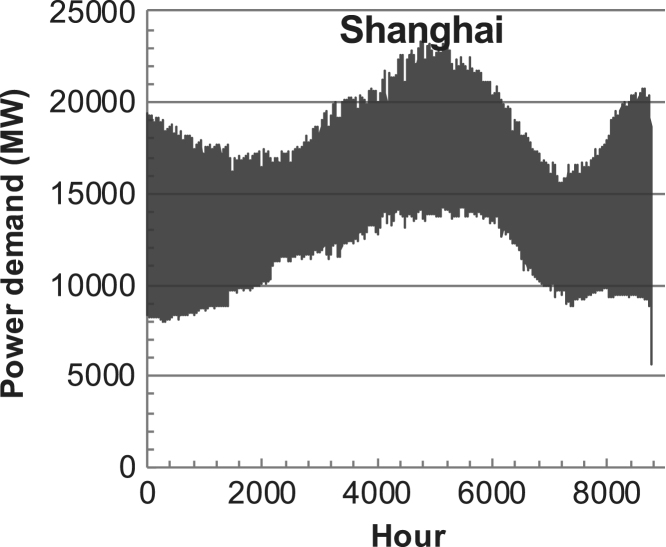
Hourly power demand for Shanghai (China) over a year (data with 1-h resolution).

**Fig. 38:**
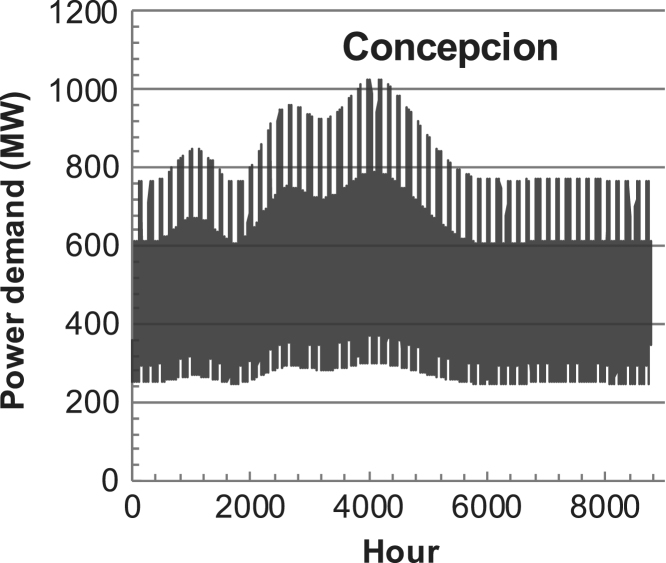
Hourly power demand for Concepcion (Chile) over a year (data with 1-h resolution).

**Fig. 39:**
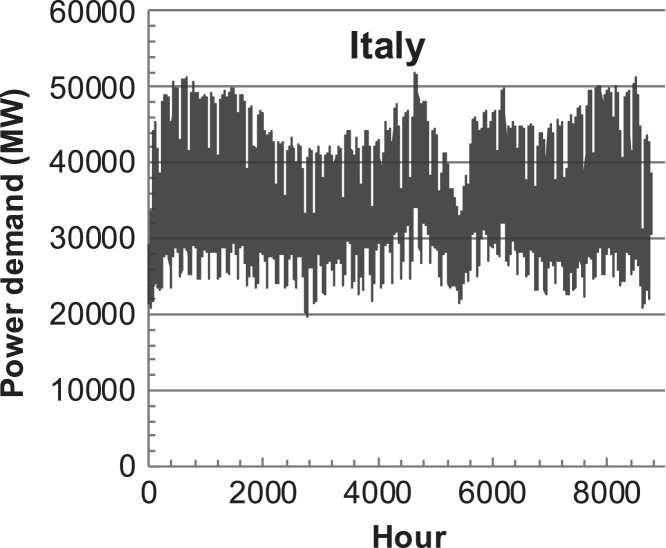
Hourly power demand for Italy over a year (data with 1-h resolution).

**Fig. 40:**
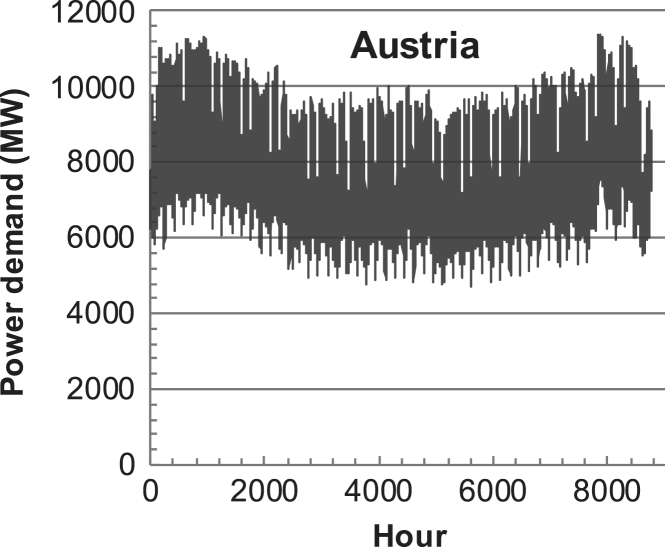
Hourly power demand for Austria over a year (data with 1-h resolution).

**Fig. 41:**
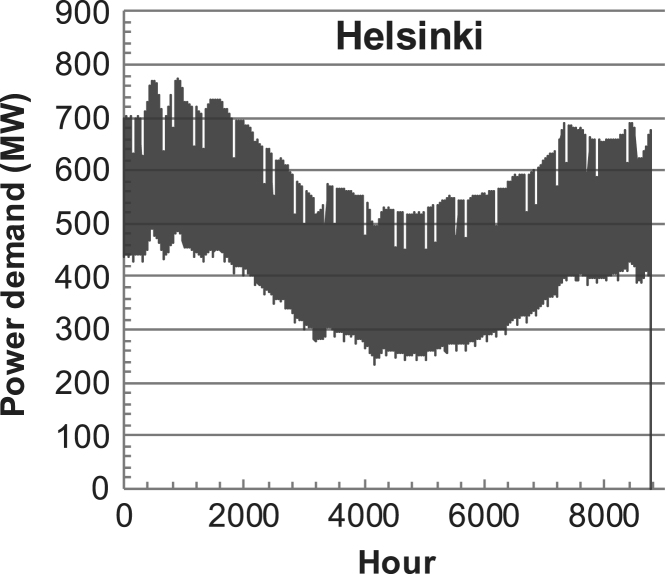
Hourly power demand for Helsinki (Finland) over a year (data with 1-h resolution).

**Fig. 42:**
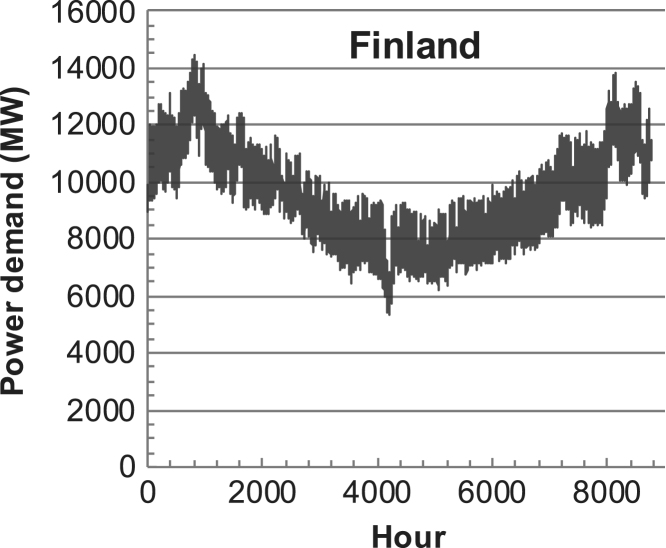
Hourly power demand for Finland over a year (data with 1-h resolution).

**Fig. 43:**
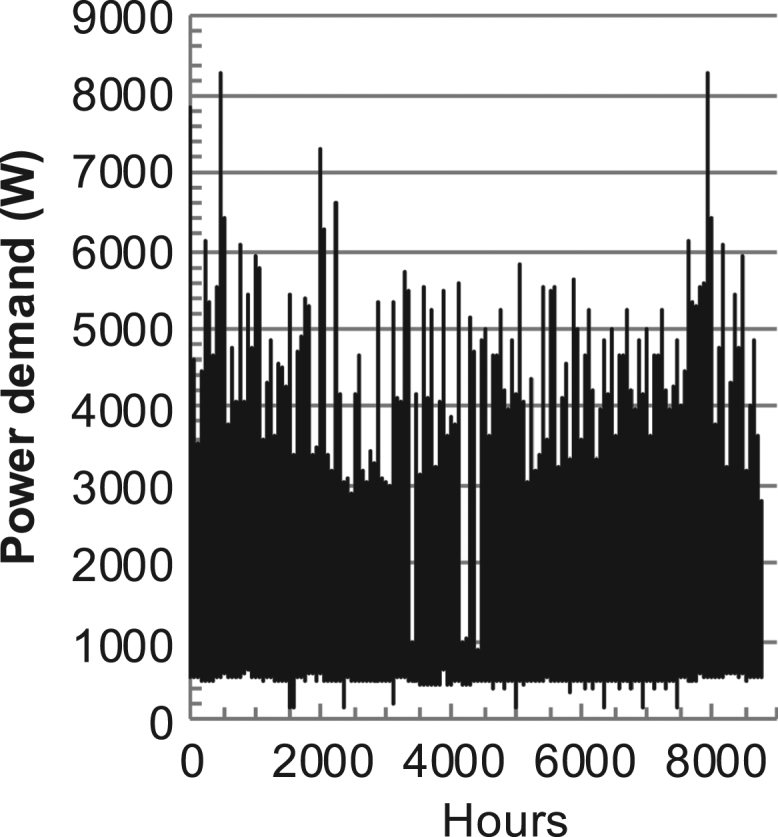
Hourly power demand for a building in Helsinki (Finland) over a year (data with 1-min resolution).

**Fig. 44:**
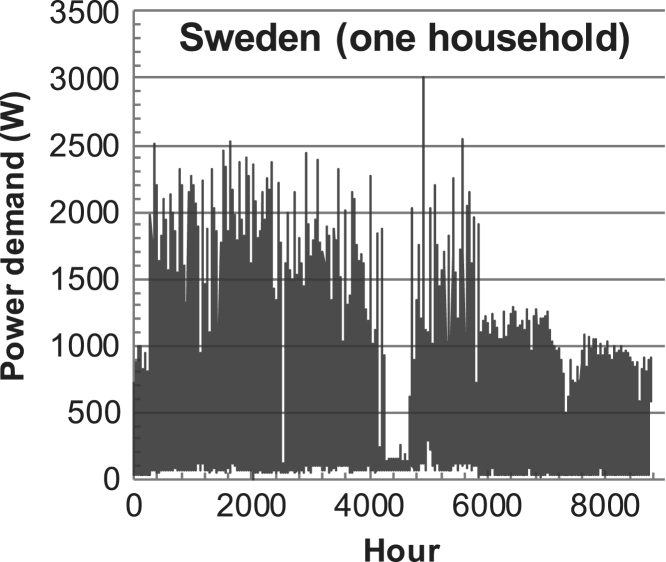
Hourly power demand for a household (L1) in Sweden over a year (data with 10-min resolution).

**Fig. 45:**
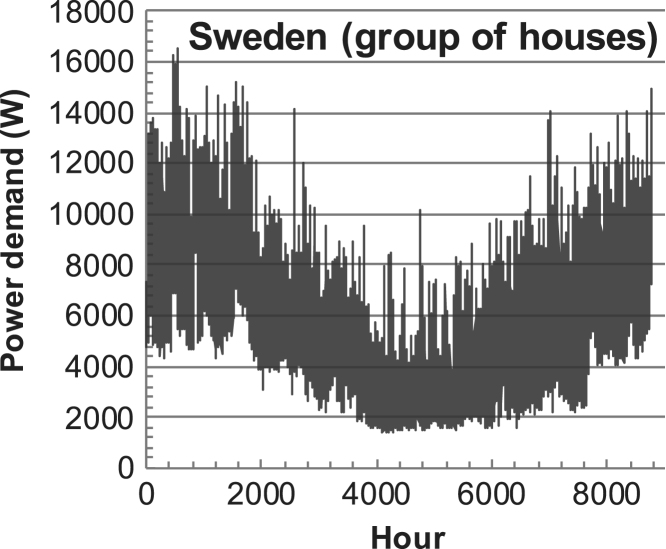
Hourly power demand for a group of buildings (L2) in Sweden over a year (data with 10-min resolution).

## 2 Experimental design, materials, and methods

The details of the data sets are described in Table 1. The location of the site is indicated for each data set and the sources for raw data. Different time scales (short-long), geographical range (north-south), spatiality (building-city-country), and climate zones (cold-hot) are covered. The range of the data is typically one year with 1-hour resolution, but for a few data sets the time resolution is 1 min or 10 min.

**Table 1:** Data sets for PV output and power demand (load).

**Type of data**	**Location**	**Resolution**	**Range**	**Reference**
PV output	60 N (Helsinki, Finland)	1 min	1 year	[2]
		1 h	1 year	
		1 h	25 years	
	59 N (Norrköping, Sweden)	10 min	1 year	[3]
	26 N (Dhahran, Saudi-Arabia)	1 h	1 year	[4]
	28 N (Delhi, India)	1 h	1 year	[5]
	31 N (Shanghai, China)	1 h	1 year	[6]
	36 S (Concepion, Chile)	1 h	1 year	[7]
	42 N (Rome, Italy)	1 h	1 year	[8]
	48 N (Vienna, Austria)	1 h	1 year	[8]
Demand profiles	Eastern Saudi-Arabia (region)	1 h	1 year	[4]
	Delhi (city)	1 h	1 year	[12]
	Shanghai (city)	1 h	1 year	[12]
	Concepion (city)	1 h	1 year	[12]
	Italy (national)	1 h	1 year	[12]
	Austria (national)	1 h	1 year	[9]
	Helsinki (city)	1 h	1 year	[12]
	Finland (national)	1 h	1 year	[10]
	Helsinki (building)	1 min	1 year	[11]
	Sweden (buildings; L1, L2)	10 min	1 year	[3]

The PV output data is for a 30-degree tilted surface orientated to the south. The 25-year solar data set for Helsinki (Finland) is for years 1973–1997 and this data is for a horizontal surface only. Except for one data set (Helsinki) with 1-min resolution, all other data is hourly.

The power demand (load) data is for hourly demand over 1 year, except for one dataset with 1-min resolution for a building in Helsinki, and for two data sets in Sweden (L1,L2) with 10-min resolution.

The household, building, regional, and national load profiles (Eastern Saudi-Arabia, Italy, Austria, Finland, Helsinki/building, Sweden/buildings L1, L2) are based on measured data, whereas the load profiles of the cities are based on simulated spatiotemporal load profiles (Conception/Chile, Delhi/India, Helsinki/Finland, Shanghai/China). The method employed to generate these hourly profiles is explained in [7], [12]. The city, regional, and national load profiles are aggregated demands of the whole electricity sector.

Sweden load L1 (see Table 1) represents a single household load based on appliances and lighting, whereas Sweden L2 is a block of houses with a stronger seasonal component (electric heating).

The PV output is calculated with a simulation tool (ALLSOL) [13] from measured solar radiation (reference to solar data given in Table 1) and ambient temperature data. For PV technology, a standard Si-module is used. The PV-output is modeled as temperature dependent.
